# Comparative Investigation of the Spectroscopic Behavior Based on High-Concentrated Solution in Nitrogen and Air Atmospheres

**DOI:** 10.3390/ijms241612629

**Published:** 2023-08-10

**Authors:** Xuefei Zhang, Ning Duan, Linhua Jiang, Fuyuan Xu, Weidong Li

**Affiliations:** 1 School of Materials Science and Engineering, Anhui University of Science and Technology, Huainan 232001, China; zhangxuefeifd@foxmail.com; 2 State Key Laboratory of Pollution Control and Resources Reuse, College of Environmental Science and Engineering, Tongji University, Shanghai 200092, China; fuyuanxu@tongji.edu.cn (F.X.); liweidong2020@163.com (W.L.); 3 Shanghai Institute of Pollution Control and Ecological Security, Shanghai 200092, China

**Keywords:** atmosphere change, direct spectral detection, high concentration, accuracy, attenuation factor

## Abstract

In order to accurately obtain photometric information of high concentration SO_4_^2−^ and other substances in the process industry, the spectroscopy behavior of SO_4_^2−^, S^2−^, Ni^2+^ and Cu^2+^ in air and nitrogen atmosphere was compared based on the UV-visible spectrophotometer with a nitrogen replacing the oxygen. Different from Ni^2+^ and Cu^2+^, the accuracy of SO_4_^2−^ and S^2−^ in the ultraviolet region was effectively improved by using a nitrogen atmosphere (P detection results were regressed within the limited standard range, RE < 5%). The nitrogen atmosphere suppressed the additional light attenuation caused by its absorption of ultraviolet rays by isolating oxygen and was also reflected in the decrease in the degree of red shift of the characteristic wavelength for SO_4_^2−^ with increasing concentration. Therefore, the detection results of SO_4_^2−^ showed an effective improvement in sensitivity. Nevertheless, according to the complementary experimental results and theoretical calculations, in addition to oxygen absorption, the low detection accuracy of SO_4_^2−^ high concentration is also attributed to the reduction of the energy required for electronic excitation per unit group caused by the interaction between SO_4_^2−^ groups, resulting in a deviation of the C-A curve from linearity at high concentrations. The influence of this intermolecular force on the detection results is far more important than oxygen absorption. The research can provide reliable theoretical guidance and technical support for the pollution-free direct measurement of high-concentration solutions in the process industry and promote the sustainable development of the process industry.

## 1. Introduction

The conflict between industrial development and environmental pollution has caused worldwide concern [1]. The process industry has promoted the rapid growth of the global economy; however, the low resource utilization rate and environmental pollution associated with the process industry are the key difficult issues faced by all countries, which restrict local environmental protection and economic development [2,3]. The feedback, adjustment and control of mass concentration balance in the process industry based on real-time monitoring technology are the key to addressing the low resource utilization rate and pollution of the process industry [4,5]. The existing national standard methods for substance detection have been widely used in the fields of environmental treatment [6,7], water quality detection [8,9,10], the biopharmaceutical industry [11,12] and the food industry [13,14]. However, these national standard methods still require complex pretreatment processes (filtration, digestion [15], extraction [16], oxidation/reduction, complexing/coloring [17], etc.) for high-concentration samples to be tested; as a result, the detection results lag behind the technical process of production seriously [18,19], and the real-time monitoring of mass concentration balance in the liquid phase cannot be realized. Therefore, the nonpolluting direct detection of high-concentration solutions in the process industry is the key to achieving sustainable industrial development and clean production.

Most process industries such as pharmaceutical, mining and wet metallurgy contain high concentrations of sulfate [20,21,22,23], metal cations [24,25,26] and non-metal ions [27,28]. As an example, the pharmaceutical industry produces antibiotic wastewater, in which the sulfate concentrations reach tens of thousands of milligrams escalation. In addition, the diversity of material occurrence state is another characteristic of the process industrial liquid state. In addition to an ionic state (e.g., As^3+^, As^5+^, Cu^2+^, Ni^2+^, etc.), there are diverse occurrence states such as group state (e.g., SO_4_^2−^, AsO_3_^3−^ and AsO_4_^3−^), solid state (e.g., CuS and Cu_2_S), as well as gas state (e.g., H_2_S and SO_2_) in process industry. Furthermore, the reaction rate of the process industry is fast; taking the copper and arsenic separation process as an example, it takes about 40 s from the input of materials to the termination of the reaction process. In view of all the above-mentioned reasons, spectrophotometry based on light absorption is an alternative technique for nonpolluting direct detection of process industrial liquids, because it is simple, fast and environment friendly. However, the nonpolluting direct detection of high-concentration solution based on spectrophotometry is still limited by a low upper detectable concentration limit and low detection accuracy. Many researchers have carried out detailed concentration detections of sulfate, metal cations and nonmetallic ions. For example, Lima et al. determined 0.1~1.5 mg/L sulfate concentration by using the spectrophotometric method [29]. Zhou et al. quantitatively measured the concentrations of 0.5~5 mg/L copper, 0.3~3 mg/L cobalt and 0.6~6 mg/L nickel ions in the technical process of zinc hydrometallurgy [30]. Gürkan et al. determined 4~450 μg/L inorganic arsenic content in spring water, beverage and food by using turbidity point extraction combined with UV-Vis spectrophotometry [31]. However, the upper concentration limits of the available laboratory detection studies are still far below the actual industrial levels.

Our preliminary research found that many substances in the process industry have characteristic absorption wavelengths within the ultraviolet region [32]. The detection of target substances within the ultraviolet region (e.g., SO_4_^2−^, AsO_4_^3−^, Zn^2+^, Pb^2+^, S^2−^, etc.) also faces interference caused by ultraviolet attenuation. Specifically, UV light with a wavelength lower than 240 nm is easily absorbed by oxygen in the air along the optical transmission path, resulting in attenuation of light intensity that is not attributable to the sample absorption [33,34]. Wang et al. pointed out the extent of the absorption of ultraviolet light by different concentrations of oxygen in the environment [35]. Ai et al. proved that the accurate determination of sulfate was interfered with by different levels of the absorption of ultraviolet light by oxygen [36]. Nitrogen has proved to be an ideal protective gas for measurements within the ultraviolet region. However, most research has ignored the differences in light absorption properties of elements in air and nitrogen atmospheres. The key to realizing the rapid direct detection of high-concentration liquid flow systems in the process industry is to accurately obtain the spectral information on the target substance at a specific wavelength. Therefore, it is very important to compare the spectral behaviors of high-concentration solutions in two different atmospheres: air and nitrogen.

In this research, four kinds of substances with characteristic wavelengths in different bands were selected within the full band range of 180~900 nm: SO_4_^2−^ (180~200 nm), S^2−^ (200~300 nm), Ni^2+^ (300~500 nm) and Cu^2+^ (600~900 nm), so as to conduct in-depth research on the changes of spectral characteristics of substances in air and nitrogen atmospheres and the influences on the detection results. The influence of the absorption of ultraviolet light by oxygen on the detection of substances within the ultraviolet region was verified by replacing air with nitrogen. Through the relative error and the spiked recovery percentage, the accuracy of the detection results in different atmospheres was compared. The comparative analysis of spectral behaviors revealed that the synergistic effect of the absorption of ultraviolet light by oxygen and the intergroup force of SO_4_^2−^ led to the deviation of the detection of high-concentration SO_4_^2−^ and the deviation of the C-A curve from linearity. The DFT calculation verified the variation of the intergroup force of SO_4_^2−^ with group concentration and its influence on the energy required for electron excitation of groups. This research revealed the changes in the spectral behaviors of the high-concentration groups and the mechanism affected by the concentration, thus providing technical support for the nonpolluting real-time monitoring of high-concentration solutions in the process industry.

## 2. Results and Discussion

### 2.1. Comparison of Measurement Errors in Air and Nitrogen Atmospheres

**Figure 1:**
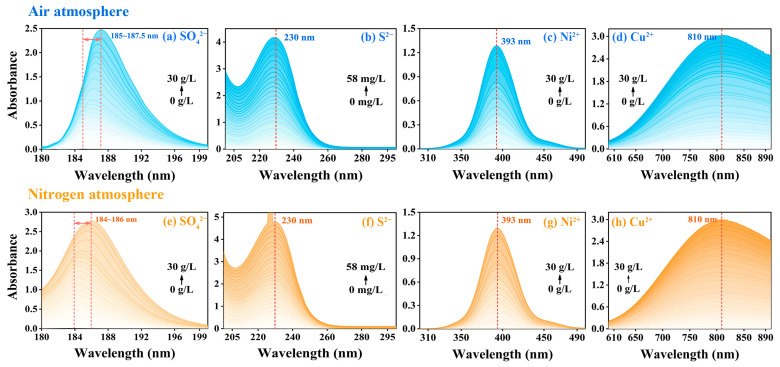
Spectral curves of SO_4_^2−^, S^2−^, Ni^2+^ and Cu^2+^, respectively in air atmosphere ((**a**–**d**), blue curve) and nitrogen atmosphere ((**e**–**h**), yellow curve) when b = 5 mm.

In order to quantify the influence of concentration changes of different substances on the characteristics of their spectral curves, four target substances located within the deep ultraviolet region (SO_4_^2−^), ultraviolet region (S^2−^), visible region (Ni^2+^) and near-infrared region (Cu^2+^) were selected for spectrophotometric detection under both air and nitrogen atmospheres. When the optical path was fixed at 5 mm, the absorption intensity of SO_4_^2−^ (Figure 1a,e), S^2−^ (Figure 1b,f), Ni^2+^ (Figure 1c,g) and Cu^2+^ (Figure 1d,h) showed an increasing trend along with the increase of concentration. Among them, due to its high molar absorption coefficient, S^2−^ could reach a high absorption intensity in a low concentration range (0~58 mg/L), even beyond the upper limit of instrument detection (as shown in Figure 1b,f). It was noted that the characteristic absorption wavelength of S^2−^, Ni^2+^ and Cu^2+^ did not shift with the increase of concentration, while the characteristic absorption wavelength of SO_4_^2−^ showed a redshift phenomenon along with the increase of the concentration of SO_4_^2−^.

**Figure 2:**
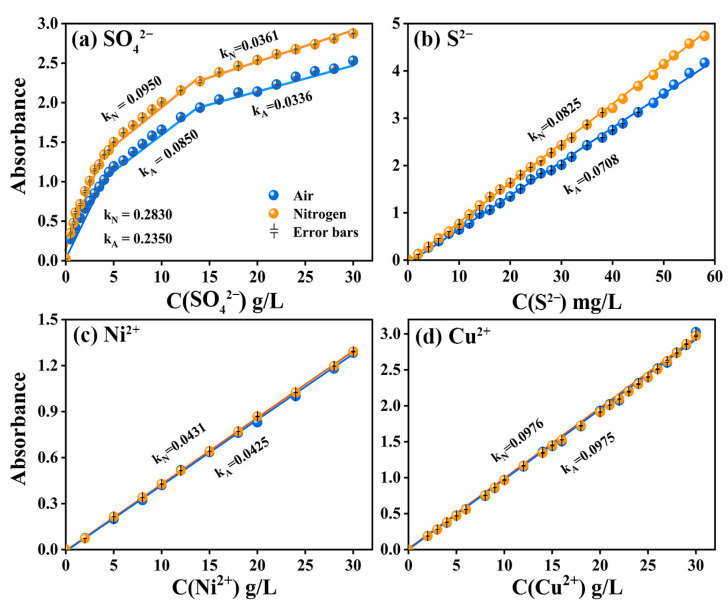
C-A curves of SO_4_^2−^, S^2−^, Ni^2+^ and Cu^2+^, respectively in air (blue curve) and nitrogen atmosphere (yellow curve) when b = 5 mm.

As for the spectral curve, the absorption intensity at the spectral peak varied most significantly with the concentration. In other words, the spectral peak had a relatively higher sensitivity [37]. When the length of the optical path was constant, the intensity of the absorption of light by substances was directly proportional to the concentration of the substance [38,39]. Therefore, the concentration–absorption intensity relationship curves were established according to the spectral curves of different ion/group concentrations, as shown in Figure 2. Figure 2a shows the piecewise fitting of the C-A curve of SO_4_^2−^ based on linear correlation coefficient R^2^ ≥ 0.95. By comparing Figure 2a,b, it can be seen that the C-A curves of SO_4_^2−^ and S^2−^ rose in varying degrees due to nitrogen that isolated oxygen to avoid the absorption of ultraviolet light, which was manifested in the increase of the slope of C-A curve, while the slopes of C-A curves of Ni^2+^ and Cu^2+^ before and after the change of atmosphere did not change significantly (Figure 2c,d). It can be inferred that the absorption of ultraviolet light by oxygen considerably interfered with the detection results within the ultraviolet region. In addition, the comparison results of baseline flatness under air and nitrogen atmospheres are shown in Figure S2. Baseline flatness presented significant noise in the UV region, which further proved that the spectrophotometric detection results are mainly affected by the absorption of ultraviolet light by oxygen. Other factors such as sampling workware, glassware and instrument functions have a relatively small impact and are not sufficient to interfere with the judgment of nitrogen improvement effect.

**Figure 3:**
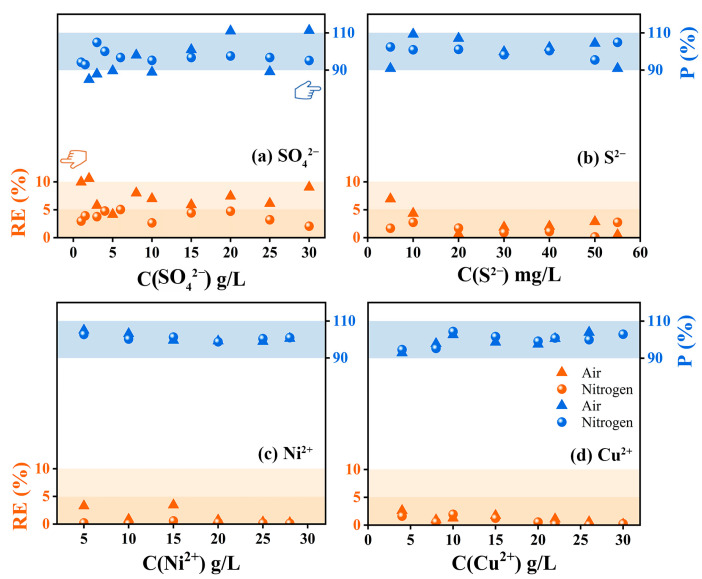
Verification of relative errors (orange) and spiked recovery percentage (blue) of SO_4_^2−^, S^2−^, Ni^2+^ and Cu^2+^ when b = 5 mm.

Meanwhile, the relative error (RE, the limited standard range should be less than 10%) and the spiked recovery percentage (P, the limited standard range should be between 90% and 110%) of the four substances in air and nitrogen atmospheres were calculated by back-calculation of ion/group concentrations according to the C-A fitting curve, and the results were shown in Figure 3. Figure 3a indicated that the relative errors of SO_4_^2−^ detection results in air atmosphere were between 5% and 10%, and the spiked recovery percentage at some concentration points exceeded the limited standard range. By using nitrogen, the relative errors of SO_4_^2−^ detection results were reduced to less than 5%, and the spiked recovery percentage came back to the limited standard range. In contrast, the spiked recovery percentages calculated for S^2−^, Ni^2+^ and Cu^2+^ under the two atmospheres were all within the limited standard range, but the spiked recovery percentages of S^2−^, Ni^2+^ and Cu^2+^ under the nitrogen atmosphere were closer to 100% (Figure 3b–d). In addition, the relative error detection results of S^2−^ in the air atmosphere were slightly higher than those in the nitrogen atmosphere (the values were all lower than 5% in the nitrogen atmosphere, and the values at some points were between 5% and 10% in air atmosphere), while the detection results of Ni^2+^ and Cu^2+^ showed good robustness.

In a word, there was a difference in the accuracy of spectrophotometric detection within the whole band region, which mainly affected the accuracy of the detection of substances within the deep ultraviolet region. By using a nitrogen atmosphere to isolate oxygen that absorbed ultraviolet light, the large deviation between detection results and true value could be effectively alleviated. The source of this error and the reason why the nonlinear SO_4_^2−^ curve appears in Figure 2 has not been studied in detail.

### 2.2. The Synergistic Influence of Detection Atmosphere and Substance Concentration on the Detection of Substances in the UV Region

**Figure 4:**
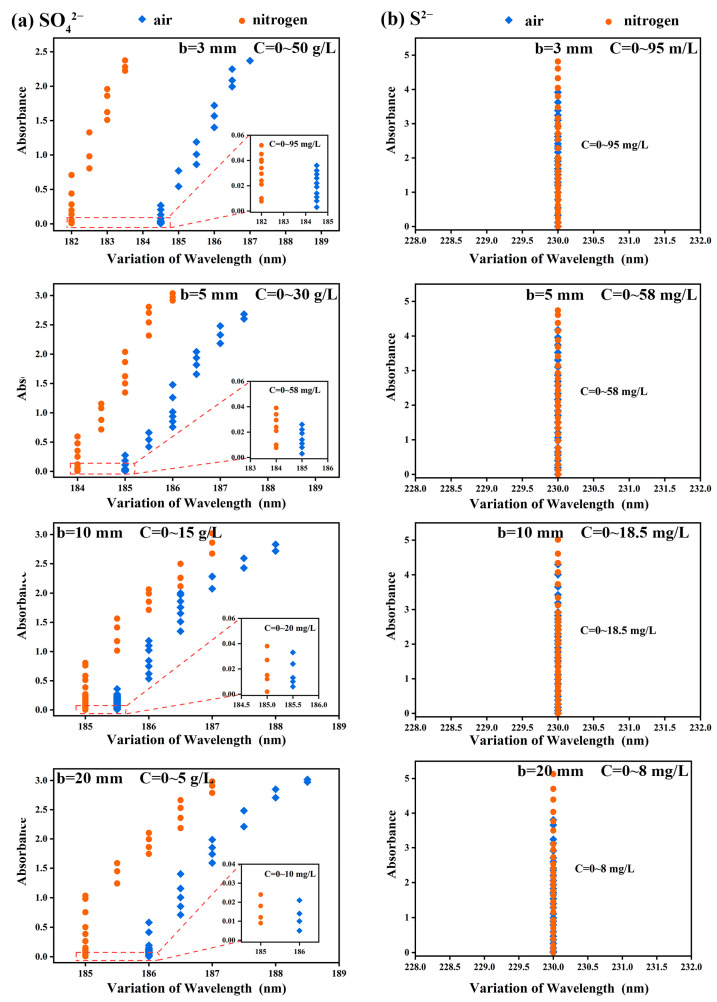
The variation law of wavelengths of (**a**) SO_4_^2−^ and (**b**) S^2−^ along with concentration under different optical paths, where the blue dot represents the air atmosphere and the yellow dot represents the nitrogen atmosphere.

Spectral curve characteristics, showing the characteristic absorption wavelength and the absorption intensity corresponding to the characteristic absorption wavelength, are used by spectrophotometric assays for the characterization and quantification of the detectors (target substances). The spectral curves measured experimentally are shown in Figure 1 and Figures S3–S5 and the statistical charts are shown in Figure 4. In this research, we focus on discussing the influencing factors of detection results within the high concentration range. The detection range of SO_4_^2−^, S^2−^, Ni^2+^ and Cu^2+^ in air and nitrogen atmosphere is 0–50 g/L, 0–95 mg/L, 0–50 g/L, 0–50 g/L and 0–50 g/L, respectively, which is much higher than the results reported in previous studies [28,30,40,41].

According to Figure 4a, the characteristic absorption wavelengths of the spectral curves of SO_4_^2−^ under different optical paths, i.e., b = 3, 5, 10 and 20 mm, in the air atmosphere (184.5~187 nm, 185~187.5 nm, 185.5~188 nm and 186~188.5 nm) were greater than those in nitrogen atmosphere (182~183.5 nm, 184~186 nm, 185~187 nm and 185~187 nm). On the contrary, the characteristic absorption wavelength of S^2−^ under optical paths of 3, 5, 10 and 20 mm did not shift with the increase of S^2−^ concentration (Figure 4b). For example, when b = 3 mm and the concentration gradually increased from 0 to 95 mg/L, the characteristic absorption wavelengths were all 230 nm, and Abs = 4.9 at this point, which reached the upper limit of instrument detection.

It was noted that the characteristic absorption wavelengths of SO_4_^2−^ did not change in the low-concentration range from 0 to 95 mg/L. However, along with the increase of the concentration of SO_4_^2−^, its characteristic absorption wavelengths showed obvious redshift. The other optical paths showed the same phenomenon as b = 3 mm. It is believed that the redshift of characteristic absorption wavelength is due to the increase of group concentration, which means that the energy required for the transition of valence electrons of polyatomic groups from low-energy-level orbit to high-energy-level orbit changes. This change in energy may be attributed to the fine structure of vibrational and rotational energy levels of valence electrons [42]. SO_4_^2−^ belongs to the inorganic oxygen-containing acid group, usually contains σ bond, π bond and n lone pair electron, and has a strong double-bond character [43], so SO_4_^2−^ is easy to generate π→π* transition after absorbing the energy of a particular wavelength. At the same time, the number of double bonds in the system increases along with the increase of SO_4_^2−^, which reduces the energy required for the π→π* transition and leads to the redshift of the characteristic absorption wavelength [42].

As shown in Figure 2a, it can be seen that when SO_4_^2−^ was at a fixed concentration, the UV absorption intensity of SO_4_^2−^ in the nitrogen atmosphere was always significantly higher than that in the air atmosphere. Specifically, when oxygen was isolated, the degree of the absorption of ultraviolet light by oxygen was significantly reduced, thus improving the absorption intensity of SO_4_^2−^. As a result, the slope of the curve in the nitrogen atmosphere was significantly higher than that in the air atmosphere. Similarly, the absorption intensity of S^2−^ also changed significantly before and after the change of atmosphere (Figure 2b). In contrast, the insensitivity of oxygen to visible light (Ni^2+^) and near-infrared light (Cu^2+^) made the detection results of substances in these wavelength ranges unaffected by the change of atmosphere (Figure 2c,d). It was also found that the C-A curves of SO_4_^2−^ with all optical paths showed obvious nonlinear changes at high concentrations (Figure 2a, Figure S6a, Figure S7a and Figure S8a). Such nonlinear changes revealed that the detection of SO_4_^2−^ solution was affected by other concentration-dependent factors besides the absorption of ultraviolet light by oxygen, which might be attributed to the acting force between oxygen-containing groups.

**Figure 5:**
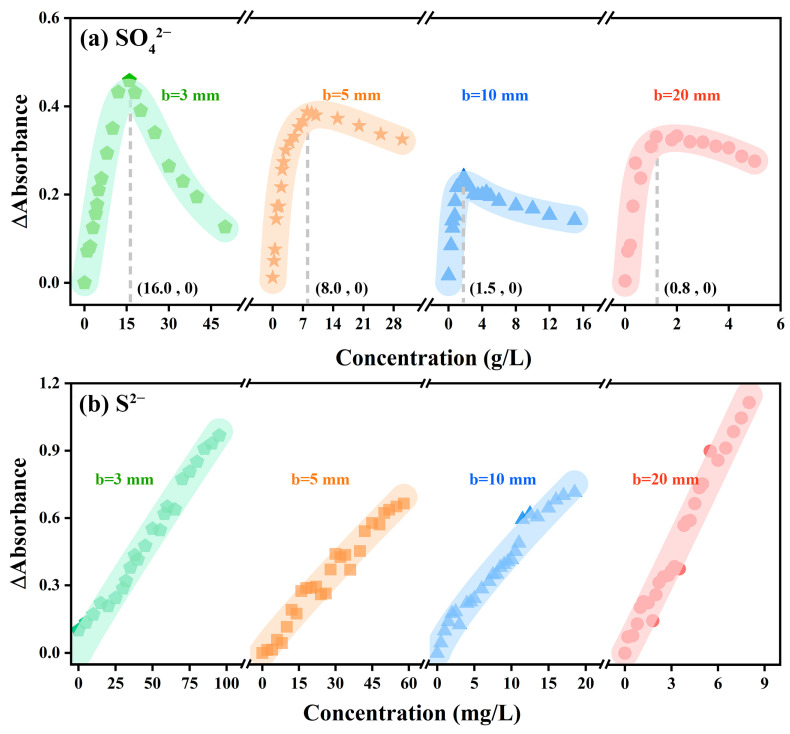
The variation trend of absorption intensity of (**a**) SO_4_^2−^ and (**b**) S^2−^ along with the concentration under different optical paths.

In order to explore the rule of the absorption intensity difference varying with the sample concentration under the two atmospheres, the variation trend of the absorption intensity of SO_4_^2−^ and S^2−^ along with the concentration under different optical paths was calculated, respectively. The results are shown in Figure 5. At the same concentration, the difference between the absorption peak intensity of the absorption spectral curve in the nitrogen atmosphere and that in the air atmosphere was defined as the variation in absorption intensity (ΔAbs). It was noted that the variation of absorption intensity of SO_4_^2−^ and S^2−^ showed an opposite trend along with the sample concentration at a high concentration. Taking b = 3 mm as an example, the variation of the absorption intensity of S^2−^ kept increasing along with the increase of the concentration of S^2−^, and the value gradually increased from 0 to 0.97 Abs. In contrast, the absorption intensity of SO_4_^2−^ increased first and then decreased along with the increase in the concentration of SO_4_^2−^. Specifically, when the concentration of SO_4_^2−^ solution was 16.0 g/L, the curve of the variation of absorption intensity showed an inflection point, which was not mentioned in previous research.

The variation of absorption intensity with concentration was determined by both external factors (change of atmosphere) and internal factors (intergroup force). In a low-concentration solution, H^+^ and SO_4_^2−^ groups could be ionized after the concentrated sulfuric acid was completely dissolved in ultra-pure water; the distance between the SO_4_^2−^ groups was much greater than the bond length of the S-O bond (149 pm) [44,45]. Thus, at low concentrations, the difference in absorption intensity between the two atmospheres was mainly attributable to the different UV absorption intensities in the two atmospheres. By using nitrogen to isolate the absorption of ultraviolet light by oxygen, the signal-to-noise ratio of the device was improved and the response of ultraviolet light intensity to SO_4_^2−^ concentration was improved.

However, along with the constantly increasing SO_4_^2−^ concentration in the system, the average distance between SO_4_^2−^ groups decreased while the intergroup forces gradually increased [40], thus affecting the charge distribution of SO_4_^2−^ groups and resulting in the weakening of SO_4_^2−^ light absorption capacity. This means that the significantly enhanced forces along with the increase of SO_4_^2−^ concentration can increase the energy required for the valence electron transition of the whole SO_4_^2−^ groups. In other words, as for the solution to be tested with a high concentration of SO_4_^2−^, UV light through the colorimeter cell was very likely to fail to enable the transition of all the valence electrons of SO_4_^2−^ groups, and the intensity increase of the absorption of UV light by SO_4_^2−^ was reduced; the decrease rate of such increase in nitrogen atmosphere was lower than that in air atmosphere, thus ΔAbs decreased at high concentrations, and the results were consistent with those in Figure 5a. In contrast, ΔAbs did not decrease with the increasing concentration of S^2−^ solution, which might be due to the low concentration of S^2−^ and the structural characteristics of S^2−^ (Figure 5b).

It was noted that along with the increase in the optical path, the concentration at the inflection point gradually decreased from 16.0 g/L (b = 3 mm) to 0.8 g/L (b = 20 mm) (Figure 5a). This is because, at the same concentration, when the light passed through a large optical path, it was more affected by the intergroup force of SO_4_^2−^, resulting in a decrease in the increase rate of absorption intensity; compared with air atmosphere, the increase rate decreased faster in the nitrogen atmosphere. Therefore, the concentration at the inflection point decreased along with the increase of the optical path.

**Figure 6:**
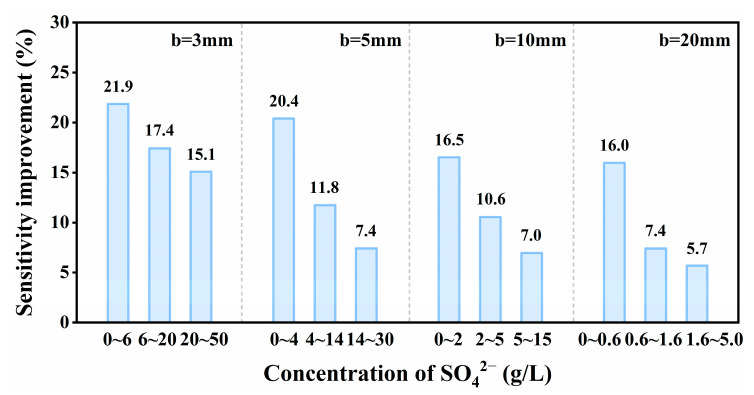
Histogram of sensitivity changes of SO_4_^2−^ in different optical paths.

The fitting slope of the C-A curve represented the sensitivity of detection results, it was used to measure the change of the absorption intensity response within a certain concentration range. The amount of variation in the sensitivity of SO_4_^2−^ at different optical lengths is shown in Figure 6. As shown in Figure 6, the spectrophotometric detection sensitivity of SO_4_^2−^ was improved by replacing air with nitrogen within different concentration ranges (the enhancing effects for b = 3, 5, 10 and 20 mm were 15.1~21.9%, 7.4~20.4%, 7.0~16.5% and 5.7~16.0%, respectively). However, as for SO_4_^2−^, the sensitivity improvement effect caused by atmosphere displacement decreased significantly in the high-concentration region. The detection of SO_4_^2−^ was affected by the synergistic interference of different detection atmospheres and the intergroup forces of SO_4_^2−^. It can be clearly concluded from Figure 6 that the sensitivity enhancement effect decreases with the increase of SO_4_^2−^ concentration at the same optical paths. Taking b = 3 mm as an example, in the low-concentration range, the intergroup force of SO_4_^2−^ was smaller. At this time, the sensitivity value measured in the nitrogen atmosphere was higher than that measured in the air atmosphere, indicating that atmosphere replacement was the reason for the largest variation of sensitivity values. However, the intergroup force of SO_4_^2−^ increased due to the further increase of SO_4_^2−^_4_ solution concentration. Therefore, the sensitivity values within the high-concentration range decreased in the two atmospheres. In addition, the sensitivity difference between the two atmospheres at high concentration was lower than that at low concentration, and the overall sensitivity improvement showed a decreasing trend along with the increase in concentration. The same is true for the analysis of other optical paths.

In a word, due to the synergistic effect of the absorption of ultraviolet light by oxygen and the intergroup force, there was a deviation in the detection of high-concentration oxygen-containing group SO_4_^2−^ within the deep ultraviolet region and the corresponding deviation of the C-A curve from linearity at high concentration, which led to the decline of the accuracy and sensitivity of the detection results.

### 2.3. Mechanism of Intergroup Interaction Affecting Electron Excitation Properties

**Figure 7:**
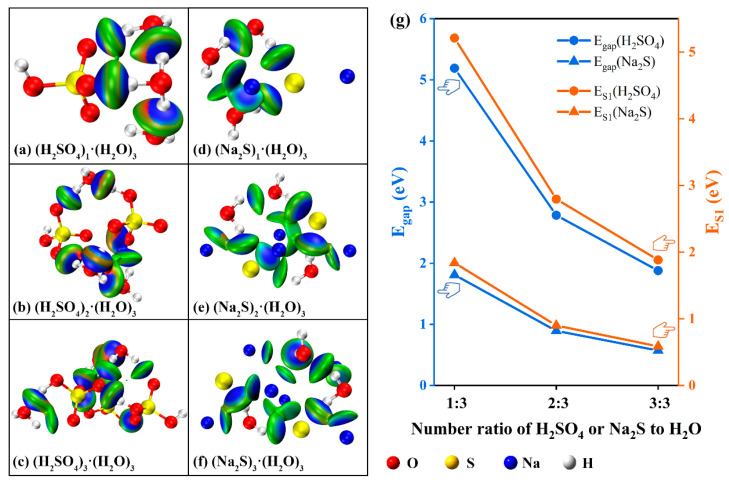
Internal forces and energy variation pattern of (H_2_SO_4_)_i_·(H_2_O)_3_ and (Na_2_S)_i_·(H_2_O)_3_ (i = 1~3) systems.

In order to further analyze the law of intergroup force increasing with group concentration in H_2_SO_4_ and Na_2_S systems and its influence on the energy required for group electron excitation, the model structures of (H_2_SO_4_)_i_·(H_2_O)_3_ and (Na_2_S)_i_·(H_2_O)_3_ (where, i = 1~3) were established, respectively [46,47], so as to simulate the intergroup forces in H_2_SO_4_ and Na_2_S solutions of different concentrations. Theoretical calculation confirmed that H_2_SO_4_ and Na_2_S both formed acting forces after being dissolved in an aqueous solution, the strength of the acting forces was shown in the blue-green ellipse in Figure 7a–f. The intergroup forces were quantified by energy gap (E_gap_) and first excited state energy (E_S1_), the specific values were shown in Figure 7g and Table 1. The energy gap (E_gap_) was defined as the difference between the LUMO orbital energy value and the HOMO orbital energy value of a single group. The first excited state energy (E_S1_) represented the energy required for valence electrons to be excited from the ground state to the first excited state and was closely related to the group’s energy gap (E_gap_).

As the ratio of (H_2_SO_4_)_i_·(H_2_O)_3_ increased from 1:3 to 3:3, the acting force on (H_2_SO_4_)_i_·(H_2_O)_3_ within the system increased from 5.19 eV to 5.64 eV according to calculation (Table 1). However, as shown in Figure 7g, the acting force on a single SO_4_^2−^ was reduced (from 5.19 eV to 1.88 eV, 1.88 eV = 5.64 eV/3). At the same time, the energy required by the valence electron of a single SO_4_^2−^ to be excited from the ground state to the first excited state (E_S1_) also decreased from 5.21 eV to 1.88 eV, confirming that the energy required by the electron excitation of high-concentration SO_4_^2−^ decreased along with the increase of its concentration, which was consistent with the experimental results. Therefore, the characteristic absorption wavelength of the absorption curve showed a redshift along with the increase of concentration, this was because the characteristic wavelength of the detected substance was inversely proportional to the energy required for its valence electron excitation. In addition, the trend that the energy required for electron excitation of a single SO_4_^2−^ decreased along with the increase of concentration also led to the slowdown of the characteristic absorption peak intensity along with the increase of the concentration of SO_4_^2−^. Therefore, the C-A curve deviated from linearity in the high-concentration region. It was noted that Na_2_S in aqueous solution showed similar concentration-dependent changes in intergroup forces. However, compared with H_2_SO_4_ (ΔE_gap_ and ΔE_S1_ were 3.31 eV and 3.33 eV, 5.19 eV − 1.88 eV = 3.31 eV, 5.21 eV − 1.88 eV = 3.33 eV), the ΔE_gap_ and ΔE_S1_ variations of Na_2_S were only 1.24 eV and 1.05 eV, respectively, indicating that the acting force of Na_2_S had a low correlation with its concentration. Therefore, the absorption curve of Na_2_S at low concentrations did not show the shift of characteristic absorption wavelength and the attenuation of characteristic absorption peak strength.

Meanwhile, the model structure of (Na_2_SO_4_)_i_·(H_2_O)_3_ (where i = 1~3) was also established to analyze the influence of cations on the energy required for electron excitation of SO_4_^2−^. As shown in Figure S9 and Table 1, similar to H_2_SO_4_, Na_2_SO_4_ in aqueous solution also showed a concentration-dependent evolution trend of intergroup forces, which resulted in the energy required for electron excitation of a single SO_4_^2−^ decreasing with the increase of concentration (from 4.07 eV to 1.30 eV). It was found that the variation of the characteristic value of the absorption curve of H_2_SO_4_ with concentration was mainly caused by the change in the energy required for the electron excitation of the oxygen-containing group SO_4_^2−^.

## 3. Materials and Methods

### 3.1. Sample Preparation

At room temperature, a certain amount of concentrated H_2_SO_4_ (analytically pure, 98%, Sinopharm Chemical Reagent Co., Ltd., Beijing, China), Na_2_S·9H_2_O (analytically pure, Shanghai Macklin Biochemical Co., Ltd., Shanghai, China), NiSO_4_·6H_2_O (analytically pure, Sinopharm Chemical Reagent Co., Ltd., Beijing, China) as well as anhydrous CuSO_4_ (analytically pure, Sinopharm Chemical Reagent Co., Ltd., Beijing, China) samples were accurately weighed. They were separately configured into SO_4_^2−^ stock solution (50 g/L), S^2−^ stock solution (100 mg/L), Ni^2+^ stock solution (50 g/L) and Cu^2+^ stock solution (50 g/L) for use. The above-mentioned stock solutions were diluted into SO_4_^2−^ (0~50 g/L), S^2−^ (0~95 mg/L), Ni^2+^ (0~50 g/L), Cu^2+^ (0~50 g/L) solutions with different concentration gradients for test.

In the preparation process, all solutions should be prepared with ultra-pure water (electrical resistivity >18.2 MΩ·cm, Milli-Q, Merck KGaA, Darmstadt, Germany), so as to avoid the interference of other impurity ions during the experiment process.

Volumetric flasks, beakers, pipettes, glass rods, sub-packed solution bottles and other instruments to be used in the experiment process should be washed with ultra-pure water and dried for use.

### 3.2. Instruments Construction and Measurements

The configuration of the whole set is shown in Figure S1. The specific data related to the instrument can be referred to in Table 2 and reference [48].

After 30 min preheating, four optical paths (b = 3 mm, b = 5 mm, b = 10 mm and b = 20 mm) were used to measure SO_4_^2−^, S^2−^, Ni^2+^ and Cu^2+^ solutions with different concentration gradients in air and nitrogen atmospheres, respectively. The parameters were set as follows in both air and nitrogen atmospheres: continuous and un-delayed spectral scanning mode; the range of 180~900 nm should be scanned at the interval of 0.5 nm; the spectral bandwidth should be set as 2 nm; ultrapure water solution should be selected as the reference solution during measurement. The results of the three detections were averaged as the final detection result.

### 3.3. Performance Indicators

#### 3.3.1. Sensitivity

Lambert Beer’s law states that the absorbance of a substance is directly proportional to the length and concentration of the optical path [49]. In the actual quantitative process, sensitivity k can be obtained through the calibration curve y = kx + b.

#### 3.3.2. Accuracy

The relative error represents the deviation between the measured value and the true value, used to represent the accuracy of the test results, denoted as RE and calculated according to Equation (1).

(1) $$RE=\frac{\bar{x_{i}}-\mu }{\mu }\cdot 100\%.$$

In which, $\bar{x_{i}}$ is the average of the actual results of measuring the concentration of SO_4_^2−^ solution n times, derived from the A-C curve, in g/L, μ is the theoretical value of SO_4_^2−^ solution is determined based on the configured SO_4_^2−^ concentration, g/L, RE is the relative error between the actual measured value and the theoretical value.

The spiked recovery rate refers to the ratio of the result obtained by adding a quantitative standard substance to a sample matrix without the tested substance and analyzing it according to the sample processing steps to the theoretical value. It is also used to characterize the accuracy of test results. The recovery rate of spiking is expressed in P and calculated according to Equation (2).

(2) $$P=\frac{\mu_{a}-\mu_{b}}{m}.$$

In which, $\mu_{a}$ is the spiked concentration of SO_4_^2−^ solution, g/L, $\mu_{b}$ is the Unstandardized concentration of SO_4_^2−^ solution, g/L, m is the spiked amount of SO_4_^2−^ solution, mL, P is the spiked recovery.

#### 3.3.3. Precision

The relative standard deviation is used to represent the precision of the test results, expressed in RSD. According to the Technical Guidelines for the Preparation and Revision of Environmental Monitoring Analysis Method Standards (HJ 168-2010), Equations (3)–(5) are used for calculation.

(3) $$\bar{x}=\frac{\sum_{k=1}^{n}x_{k}}{n},$$

(4) $$S_{i}=\sqrt{\frac{\sum_{k}^{n}{\left(x_{k}-\bar{x}\right)}^{2}}{n-1}},$$

(5) $$RSD=\frac{S_{i}}{\bar{x}}\cdot 100\%.$$

In which, $x_{k}$ is the absorbance test result of SO_4_^2−^ solution, n is the number of measurements, $\bar{x_{i}}$ is the Measure the average value of the absorbance test results of n times of SO_4_^2−^ solution, $S_{i}$ is the Standard deviation of absorbance test results for SO_4_^2−^ solution  andRSD is the relative standard deviation of the absorbance test results of SO_4_^2−^ solution.

### 3.4. Computational Details

The structure of (H_2_SO_4_)_i_·(H_2_O)_3_ and (Na_2_S)_i_·(H_2_O)_3_ with different proportions (i = 1~3) was established to simulate H_2_SO_4_ and Na_2_S solutions with different concentrations. Gaussian 09 software (Gaussian 09W) was used for all calculations, and the PBE0 function was adopted, under the PBE0/6-311++G(d, p) level [50], the TD-DFT method was adopted to calculate the parametric law of difference in the intermolecular forces between H_2_SO_4_ and H_2_SO_4_, H_2_SO_4_ and H_2_O, Na_2_S and Na_2_S as well as Na_2_S and H_2_O systems that varied with the concentration of substances [51]. The analysis was performed by using the Multiwfn 3.8 (dev) code [52], and the results were calculated by using the Visual Molecular Dynamics (VMD 1.9.3) software [53,54].

## 4. Conclusions

Based on a nitrogen-isolated oxygen ultraviolet-visible spectrophotometer, this study quantitatively analyzes the changes in spectral characteristics, accuracy and sensitivity of four substances (SO_4_^2−^, S^2−^, Ni^2+^ and Cu^2+^) with characteristic wavelengths located in different wavelengths under air and nitrogen atmospheres.

The experimental results indicate that the absorption of ultraviolet light by nitrogen insulation reduces the attenuation of additional light, leading to a regression of the P values of SO_4_^2−^ and S^2−^ within the limited standard range of 90 to 110%, with RE values below 5%. While there was no significant change in the P and RE values of Ni^2+^ and Cu^2+^, the analysis of spectroscopy behavior revealed that with the increase of SO_4_^2−^ concentration, the intergroup force significantly interfered with the accuracy of SO_4_^2−^ detection results in the high concentration range. The synergistic effect of oxygen absorption of ultraviolet radiation and intermolecular forces leads to bias in the detection results of oxygen-containing groups SO_4_^2−^ in the deep ultraviolet region. The change in the absorption intensity of SO_4_^2−^ obtained by isolating oxygen with nitrogen first increases and then decreases with the increase of SO_4_^2−^ concentration. By using nitrogen to isolate oxygen and absorb ultraviolet light, the sensitivity of SO_4_^2−^ detection results is significantly improved. The experiment revealed that the sensitivity improvement effect decreases with the increase of SO_4_^2−^ concentration under the same optical path, which is another important reason for the low detection accuracy of SO_4_^2−^.

The DFT calculation results confirm that the interaction force between the SO_4_^2−^ groups and the energy required for electron excitation in high-concentration H_2_SO_4_ significantly decreases with increasing concentration, resulting in a significant red shift in the characteristic absorption wavelength with increasing concentration. The effect of this action is much higher than that of oxygen-absorbing ultraviolet light and leads to a deviation of the C-A curve of SO_4_^2−^ in the high concentration range from linearity. The results of this study can provide reliable theoretical guidance and technical support for achieving pollution-free real-time monitoring of high-concentration liquids and promote the sustainable development and clean production of the process industry.

## Figures and Tables

**Table 1:** Values of the energy gap E_gap_ and the first excited state energy E_S1_ in (H_2_SO_4_)_i_·(H_2_O)_3_, (Na _2_SO_4_)_i_·(H_2_O)_3_ and (Na_2_S)_i_·(H_2_O)_3_ (i = 1~3) systems.

Models Structure	All Groups/Ions		Single Groups/Ions	
	E_gap_ (eV)	E_s1_ (eV)	E_gap_ (eV)	E_s1_ (eV)
(H_2_SO_4_)_1_·(H_2_O)_3_	5.19	5.21	5.19	5.21
(H_2_SO_4_)_2_·(H_2_O)_3_	5.57	5.59	2.79	2.80
(H_2_SO_4_)_3_·(H_2_O)_3_	5.64	5.65	1.88	1.88
(Na_2_S)_1_·(H_2_O)_3_	1.81	1.84	1.81	1.84
(Na_2_S)_2_·(H_2_O)_3_	1.79	1.80	0.90	0.90
(Na_2_S)_3_·(H_2_O)_3_	1.72	2.37	0.57	0.79
(Na_2_SO_4_)_1_·(H_2_O)_3_	4.06	4.07	4.06	4.07
(Na_2_SO_4_)_2_·(H_2_O)_3_	3.79	3.79	1.90	1.90
(Na_2_SO_4_)_3_·(H_2_O)_3_	3.90	3.90	1.30	1.30

Where E_gap_ = E_LUMO_ − E_HOMO_; E_S1_ represents the first excited state energy, i.e., the energy required for the valence electron to transition from the ground state to the first excited state and is closely related to the E_gap_ of groups.

**Table 2:** List of specific component names, models and manufacturers.

Partical Name	Component Name	Model and Manufacturer
Instrumentation	UV-visible spectrophotometer	T10CS, Beijing Persee General Instrument Co., Ltd., Beijing, China
Instrument external cover	1200 mm × 850 mm × 437 mm	Organic glass
Control nitrogen flow into the cover	1 M.F.C. (the measuring range was 0~30 L/min)	S48-32/HMT, HORIBA Precision Instruments (Beijing) Co., Ltd., Beijing, China
Control nitrogen flow into light source system	1 M.F.C. (the measuring range was 0~10 L/min)	
Control nitrogen flow into sample chamber	1 M.F.C. (the measuring range was 0~5 L/min)	
Control nitrogen flow into data receiving areauk	1 M.F.C. (the measuring range was 0~5 L/min)	
Monitor the nitrogen flow at the outlet	1 M.F.C. (the measuring range was 0~30 L/min)	
Auto-injection flow cell	Peristaltic pump	BT100-2J, Baoding Longer Precision Pump Co., Ltd., Baoding, China
The rotation speed of the peristaltic pump	30 r/min	
Automatic control device	PLC	S7-1200, Siemens AG, Munich, Germany
	Upper computer	MCGSPro, Shenzhen Kunluntongtai Automation Software Technology Co., Ltd., Shenzhen, China

## Data Availability

The data presented in this study are available on request from the corresponding authors. The data are not publicly available because of the continuous research.

## Supplementary Materials

The following supporting information can be downloaded at: https://www.mdpi.com/article/10.3390/ijms241612629/s1.
