# Supplementary material for: Comparative Investigation of the Spectroscopic Behavior Based on High-Concentrated Solution in Nitrogen and Air Atmospheres
Source: Int J Mol Sci. 2023 Aug 10;24(16):12629. doi: 10.3390/ijms241612629 (PMC10454424; doi:10.3390/ijms241612629)
Supplement: Supplementary file 1 [file ijms-24-12629-s001.zip › Supplementary Information.pdf]

Supplementary Information

Comparative investigation of the spectroscopic behavior based on high-concentrated solution in nitrogen and air atmospheres

Xuefei ZHANG [a], Ning DUAN [a,b,c*], Linhua JIANG [a,b,c**], Fuyuan XU [b,c], Weidong LI [b,c]

[a] School of Materials Science and Engineering, Anhui University of Science and Technology, Huainan, Anhui, 232001, China

[b] State Key Laboratory of Pollution Control and Resources Reuse, College of Environmental Science and Engineering, Tongji University, Shanghai, 200092, China

[c] Shanghai Institute of Pollution Control and Ecological Security, Shanghai, 200092, China.

Corresponding Authors: *Ning Duan (ningduan@tongji.edu.cn)

**Linhua Jiang (jianglinhua@tongji.edu.cn)

The supplementary materials include 9 pages.

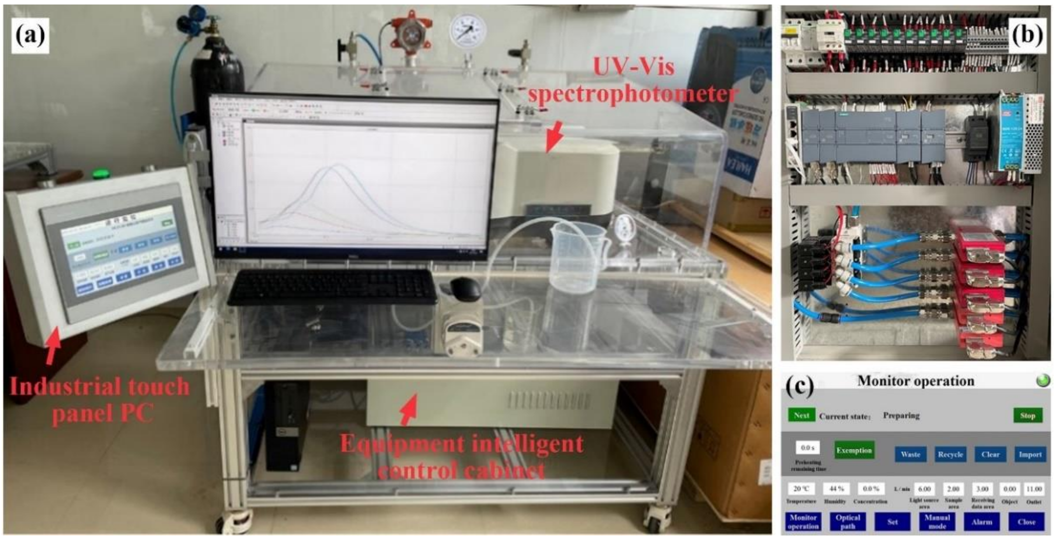

Figure S1 Configuration diagram of the whole set (a) picture of real products, (b)PLC integrated control and (c) upper computer operation monitoring interface

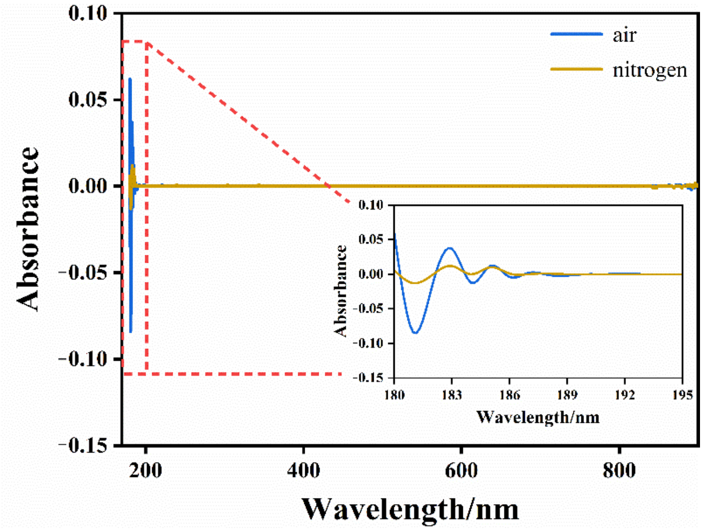

Figure S2 Comparison of baseline flatness under air and nitrogen atmospheres

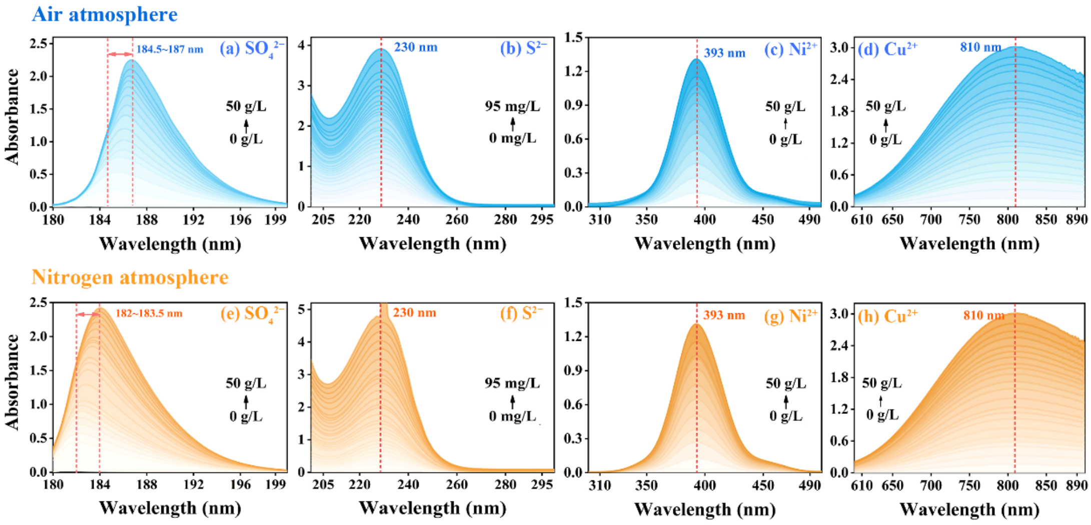

Figure S3. Spectral curves of SO_4^{2-}, S^{2-}, Ni^{2+} and Cu^{2+} respectively in air atmosphere (a~d, blue curve) and nitrogen atmosphere (e~h, yellow curve) when b=3 mm

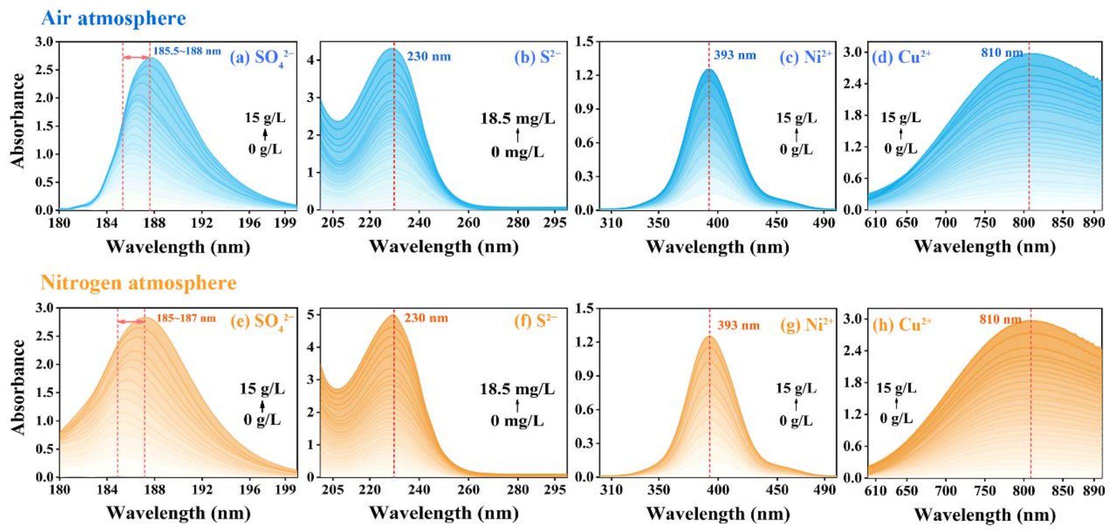

Figure S4. Spectral curves of SO_4^{2-}, S^{2-}, Ni^{2+} and Cu^{2+} respectively in air atmosphere (a~d, blue curve) and nitrogen atmosphere (e~h, yellow curve) when b=10 mm

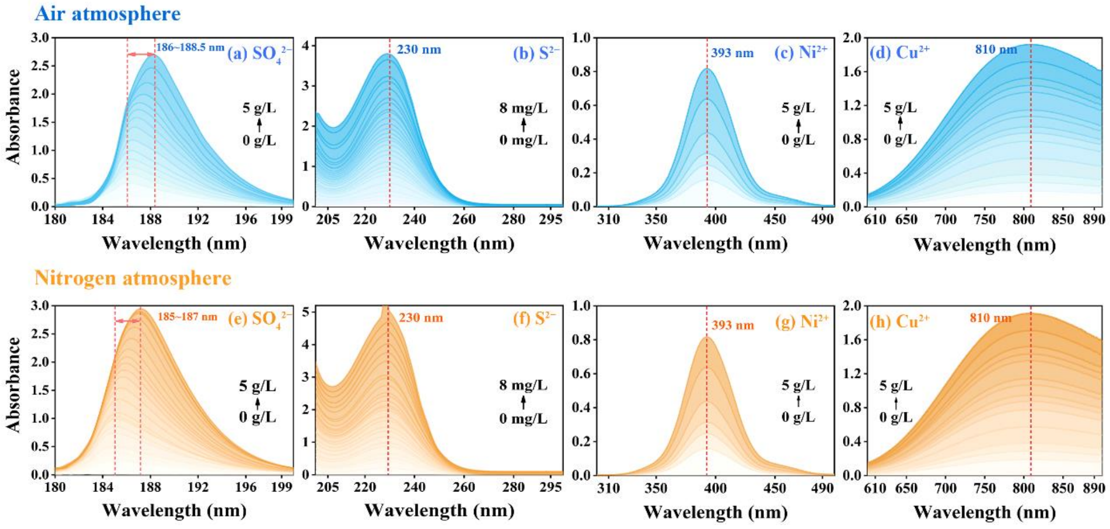

Figure S5. Spectral curves of SO_4^{2-}, S^{2-}, Ni^{2+} and Cu^{2+} respectively in air atmosphere (a~d, blue curve) and nitrogen atmosphere (e~h, yellow curve) when b=20 mm

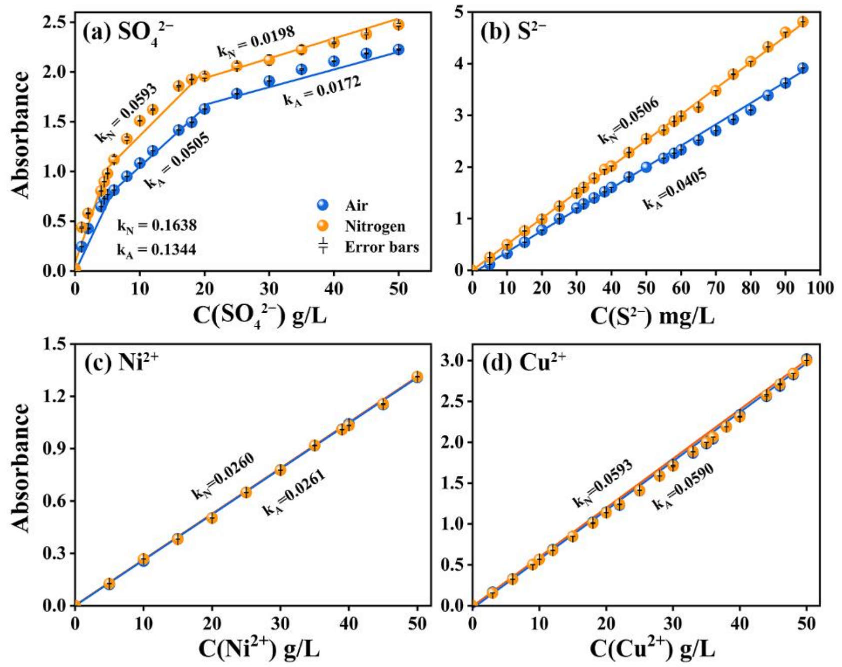

Figure S6. C-A curves of SO_4^{2-}, S^{2-}, Ni^{2+} and Cu^{2+} respectively in air (blue curve) and nitrogen atmosphere (yellow curve) when b=3 mm

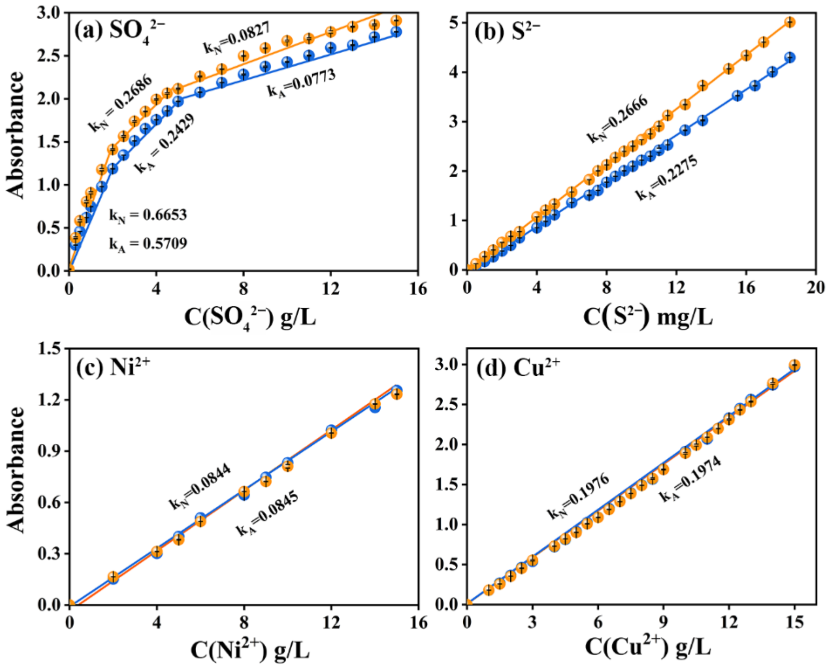

Figure S7. C-A curves of SO_4^{2-}, S^{2-}, Ni^{2+} and Cu^{2+} respectively in air (blue curve) and nitrogen atmosphere (yellow curve) when b=10 mm

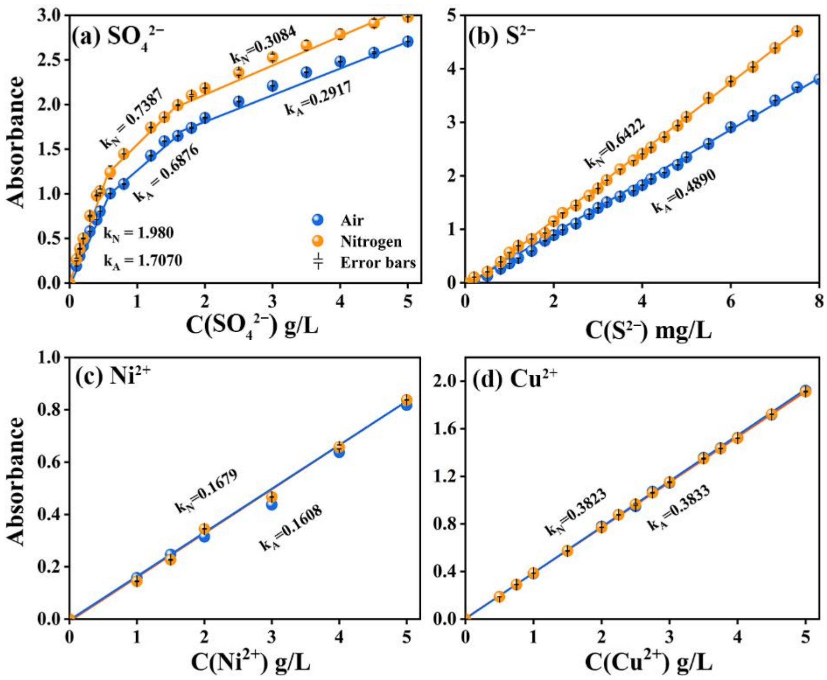

Figure S8. C-A curves of SO_4^{2-}, S^{2-}, Ni^{2+} and Cu^{2+} respectively in air (blue curve) and nitrogen atmosphere (yellow curve) when b=20 mm

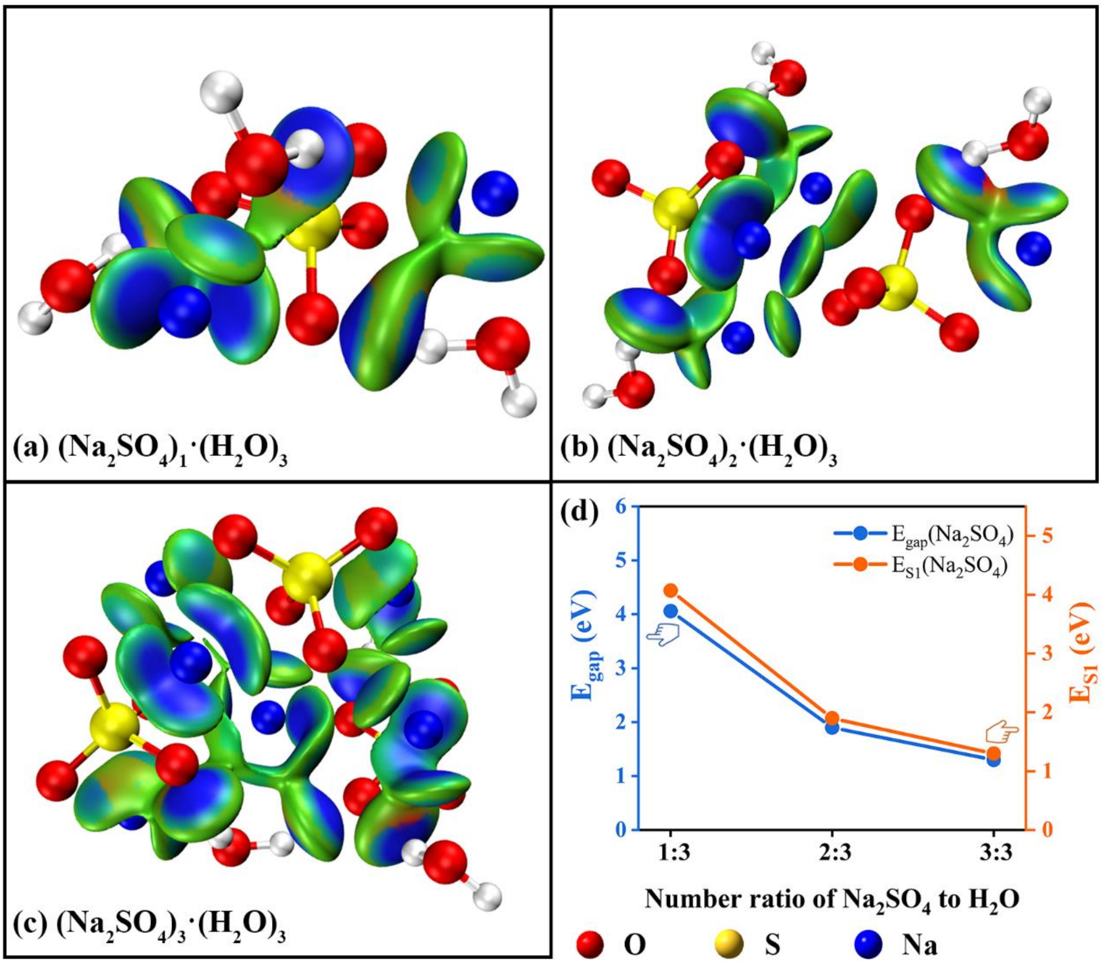

Figure S9. The variation law of forces and energy along with concentration within $(Na_2SO_4)_i \cdot (H_2O)_3$ (i=1~3) systems
